# Performing Calibration of Transmittance by Single RGB-LED within the Visible Spectrum

**DOI:** 10.3390/s20123492

**Published:** 2020-06-20

**Authors:** Daniel Carreres-Prieto, Juan T. García, Fernando Cerdán-Cartagena, Juan Suardiaz-Muro

**Affiliations:** 1 Department of Mining and Civil Engineering, Technical University of Cartagena, 30202 Cartagena, Spain; 2 Department of Information and Communications Technologies, Technical University of Cartagena, 30202 Cartagena, Spain; fernando.cerdan@upct.es; 3 Department of Electronic Technology, Technical University of Cartagena, 30202 Cartagena, Spain; juan.suardiaz@upct.es

**Keywords:** RGB-LED spectrophotometer, water pollutants, transmittance modelling, cost effective

## Abstract

Spectrophotometry has proven to be an effective non-invasive technique for the characterization of the pollution load of sewer systems, enabling compliance with new environmental protection regulations. This type of equipment has costs and an energy consumption which make it difficult to place it inside a sewer network for real-time and massive monitoring. These shortcomings are mainly due to the use of incandescent lamps to generate the working spectrum as they often require the use of optical elements, such as diffraction gratings, to work. The search for viable alternatives to incandescent lamps is key to the development of portable equipment that is cheaper and with a lower consumption that can be used in different points of the sewer network. This research work achieved the following results in terms of the measured samples: First, the development a calibration procedure that enables the use of RGB-LED technology as a viable alternative to incandescent lamps, within the range of 510 to 645 nm, with high accuracy. Secondly, demonstration of a simple method to model the transmittance value of a specific wavelength without the need for optical elements, achieving a cost-effective equipment. Thirdly, it provides a simple method to obtain the transmittance based on the combination of RGB colors. Finally its viability is demonstrated for the spectral analysis of wastewater.

## 1. Introduction

The European Union states that in order to improve indicators of compliance with the Urban Wastewater Treatment Directive, Directive 91/271, it is necessary to reduce pollution emitted through combined sewer overflows (CSOs) [1,2]. At the same time, it is recognized that there is still a lack of knowledge about how pollutants are mobilized in CSOs [3,4,5]. Overflows are closely related to rainfall, thus presenting a great variability both in their volume and in the pollutant load they transport. In addition, they can vary enormously throughout the same episode, registering pollution peaks, which in turn can be reduced to very low values in intervals of minutes [6,7,8]. These reasons make it necessary to devote significant efforts to the monitoring of overflows in order to quantify the impacts on the receiving environment, taking into account the characteristics of that environment [9,10,11]. The US DSS control policy (Clean Water Act) includes nine minimum controls to be carried out by landfill owners, including monitoring and evaluation of the contaminating load [12,13].

The monitoring of pollution in sewer networks can be at different levels, from merely taking some periodical samples to be analyzed in the lab, to the continuous monitoring of pollutants. The measurement of continuous values of turbidity, pH, conductivity, temperature, nutrients, and organic compounds through spectrophotometric probes today constitutes robust techniques that enable the mobilization of pollutants to be characterized throughout the day, on-line and continuously [4,5,14,15]. Furthermore, on-line monitoring helps in the planning of actions and infrastructures and is required in order to comply with existing legislation on wastewater treatment [16,17]. Nowadays, to address the CSOs’ pollutant dynamics, time-continuous transmittance measurements have been used with satisfactory results. Measurements are taken at several wavelengths, like those of the visible and 254 nm UV wavelengths [9,18,19]. Spectroscopy devices capable of working in the visible spectrum, such as those made of RGB-LED, will help to increase knowledge of pollutants’ movement and water quality monitoring during CSOs [20].

Cost-effective spectrophotometers based on LEDs are being developed and spread to determine wastewater pollution with high accuracy [21,22,23,24], and their comparison with transmittance calculated from classical devices based on incandescent lamps shows good agreement [25].

Low-cost RGB light-emitting diodes (RGB-LEDs) are nowadays used in the construction of simple and compact spectrophotometers for molecular absorbance measurements in analytical chemistry. For instance, RGB-LED-based sensors have been developed to measure different parameters on-line, such as the microalgae-biomass concentration, within a photo-bioreactor, with a 2% error [26]. The use of RGB allowed the study of on-line chromatic values without the cost spiraling throughout the winemaking process [27]. An RGB sensor was also used to obtain information about the color of the sample, detecting the movement of phytoplankton [28]. In the case of tap water, RGB sensors through a web cam were also utilized to control the concentration of parameters, such as ortho-phosphate and aluminum (III) [29]. In the case of wastewater, a portable RGB diode was utilized for the on-site determination of nitrite and iron in river waters [30]. The results of visible spectrophotometry in the near infrared region (NIR) using an RGB diode and a 360- to 740-nm spectrophotometer were compared when characterizing parameters, such as the ammonia concentration and electrical conductivity values [31].

RGB-LED has also been used to calculate the dense packing of bacterial cells in sample solutions in well plates, obtaining good accuracy [32]. The RGB-LED assumes the superposition of different wavelengths, as opposed to conventional spectrophotometers that apply reduced wavelengths for each measurement. This fact introduces an increased susceptibility to interference. However, several authors have shown RGB to be effective, simple, compact, and at a low cost. As stated by [33], its performance indicates that it could be suitable as a replacement for conventional spectrophotometers used in photometric analytical procedures. A loss of sensitivity will usually be encountered, and users will have to decide if this is not significant, since the accuracy continues to be high enough.

The present work includes an experimental campaign in which transmittance is measured from an RGB-LED diode through multiple water samples with different dyes and compounds that cover a broad range of transmittance. The results are compared and discussed with those obtained from a commercial spectrophotometer. An RGB-LED is operated with different equations to vary the emitted superposition of wavelengths, so as to cover the widest possible range within the visible spectrum of light, making use of the combination of the three small individual LEDs contained within the RGB-LED.

The contributions of this work are as follows: First, we developed a novelty calibration process, in order to measure transmittance values between 510 and 645 nm using a single RGB-LED, without optical devices, and with a high level of accuracy. As indicated in the study [25], 18 individual LEDs are needed to be able to analyze water samples between 510 and 645 nm; therefore, a single RGB-LED enables us to significantly reduce the cost of the equipment. Secondly, we provide a simple way to define the red, green, and blue RGB-LED intensity combinations to be used in order to measure the transmittance values that are in agreement with those obtained using the commercial equipment. The novelty lies in using the RGB combination to produce a chemical response in water samples; this can be correlated with that obtained by equipment based on incandescent lamps, where a single wavelength passes through the sample.

## 2. Materials and Methods

### 2.1. Analyzed Samples

Table 1 shows the different samples used in this research. From a total of 48 samples, 20 (S1-–S20) were used to carry out the calibration process, to ensure that the transmittance values provided by the RGB-LED are close to those provided by commercial equipment based on incandescent lamps.

In order to evaluate the suitability of the calibration models, 28 water samples were analyzed, including nine from urban wastewater of the city of Cartagena (Spain), and whose origin includes important industrial areas. These wastewater samples were collected at different points at the Cabezo Beaza wastewater treatment plant: Wastewater treatment plant inlet (S21, S43, and S44), at the Primary Settler outlet (S22, S45, and S46), and at the treatment plant outlet (S23, S47, and S48).

Likewise, the food dyes used to generate part of the calibration samples were as follows: E105 (S3), E133 (S3 and S4), E124 (S10, S13, and S14), E100 (S16), and E133 (S19)

All the samples were stored in standard 12 × 12 × 50 mm plastic test tubes of the SEOH brand [34], designed for spectrophotometry purposes.

### 2.2. Reference Equipment

In the present work, all the results were contrasted with the commercial equipment V-5000 VIS [35]. This has a working spectrum of between 325 and 1000 nm, with a bandwidth of 4 nm, which complies with the following standards:

- ISO 22891: 2013: Determination of transmittance by diffuse reflectance measurement.
- ISO 10110-9: 2016: Preparation of drawings for optical elements and systems—Part 9: Surface treatment and coating.
- ISO 7887:2011: Water Quality—Examination and determination of color.
- ISO 9001 7.6: Control of monitoring and measuring equipment.

### 2.3. Sensor Unit

To develop a cost-effective equipment, one of the main points to tackle is the sensor unit. Most commercial equipment are based on CCD (charge-coupled device) sensors [36], which can measure multiple wavelengths at the same time. However, their high price and the need for an additional elements to work (mainly an optical element), increase the price of such equipment. For this reason, and pursuing the “minimum viable solution”, i.e., one that makes use of as few elements as possible, we used an individual photodiode as a sensor, which is designed to capture a single beam of light [37,38] from a wide range of wavelengths. Once the kind of sensor to be used has been chosen, it remained to determine which one to choose, since this type of sensor is marketed with different sensitivities and spectral responses. In order to determine which properties the sensor must have in order to be valid for this type of application, we carried out a comparison between several models. To simplify the explanation, we focused the study on two of them: S1223 [39] and OSD15-E [40].

**Figure 1:**
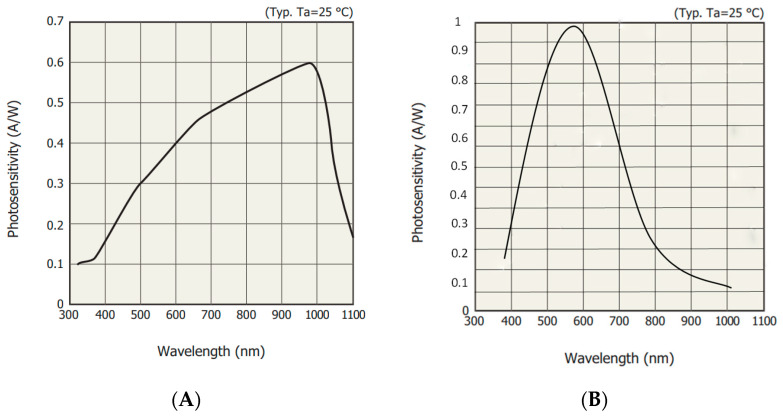
Sensitivity curves of the photodiodes (**A**) S1223 and (**B**) OSD15-E.

Figure 1 shows the spectral response of both sensors. A photodiode can have a different spectral response according to the wavelength of the incident light, that is, it will be able to detect more of some wavelengths than others, as shown in the figure.

If the same sample is traversed by a certain wavelength, for example, at 470 nm, with a certain brightness level, as shown in Figure 1, the measured value *I* (amount of detected light through the sample) will be different. The sensor OSD15-E (Figure 1B) presents at that wavelength a sensitivity over 50%, while S1223 (Figure 1A) barely manages to reach 25%. This implies that the former (OSD15-E) will be able to detect twice as much light as the latter, and therefore, the *I* value will be different. This fact could lead us to think that the best sensor to choose will be the one that is capable of capturing a greater amount of light at a certain wavelength, i.e., the one with the highest sensitivity. However, we must take into account that the transmittance $\left(T=\frac{I}{I_{0}}\right)$ is the result of the amount of light that manages to pass through the sample we seek to analyze (I), with respect to the amount of light that passes through a reference sample $\left(I_{0}\right)$, typically distilled water. In addition, we must note that if the *I* value is twice as much on the OSD15-E sensor as S1223, the $I_{0}$ value of the first one (OSD15-E) will also be twice that measured in S1223. Therefore, the transmittance relation will remain constant regardless of the type of sensor used. A series of measurements performed proved that the differences between the transmittance values provided by the two sensors in all cases were less than 4%.

Based on the tests carried out, we concluded that the sensor that has a sensitivity of at least 5% for the wavelength to be analyzed is valid for spectrophotometry applications. As we focused the study on the visible spectrum, the sensor S1223, which has a monetary cost three times lower than OSD15-E, has been chosen to carry out the analysis of the samples shown in Table 1. However, the price difference between the two is not particularly significant, so either could have been chosen without overly affecting either the price of the equipment or its accuracy.

At this point, once the sensor was chosen, it was necessary to take into account that this type of sensor generates an electrical current according to the amount of light that falls on them, which is of the order of picoAmperium. Most microcontrollers/microprocessors are only capable of measuring voltage levels within a certain range, for example, 0–3 v. Therefore, the values provided by the sensors must be converted to the voltage and amplified by a transimpedance circuit. In our case, we configured the sensor to provide a response within the 0–5 V range.

### 2.4. RGB Light-Emitting Diode (RGB-LED)

Incandescent lamps are able to generate a wide range of wavelengths on their own; however, they have an associated power consumption and high temperature issues, as well as being large in size, due to the additional elements required to operate them (overall, optical elements). Therefore, the present research work focused on determining whether a new light source could replace them in a cost-effective way.

A priori, one idea that would come to mind is to use LED technology, due to its low power consumption and lack of temperature problems. Nevertheless, for analyzing the entire spectrum would require the use of a huge number of LEDs [25], since each of them is designed to emit within a specific range of wavelengths. This would therefore result in an increase in both the size of the equipment as well as its cost.

Not all applications that make use of the spectrometry technique require the use of a full multispectral analysis but merely some specific spectral points. For instance, the analysis of chlorophyll focuses only on the 600-nm wavelength. For these reasons, in the current research work, we studied the RGB-LED as a source of light for spectrophotometry applications, in order to define how many wavelengths can be modelled by a single RGB-LED, without using optical devices; more specifically, we used the HV-5RGB25 [41], a common cathode diode, 5 mm in diameter.

**Figure 2:**
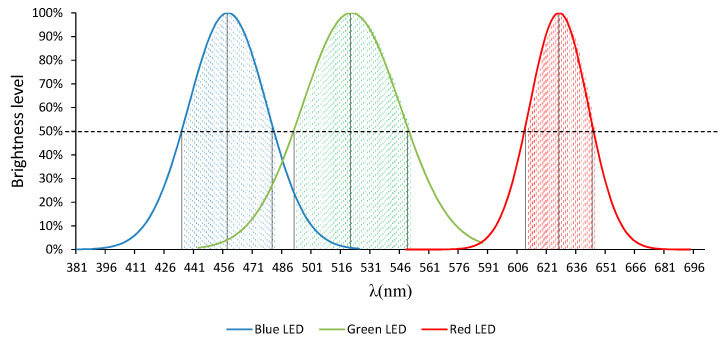
Emission spectrum RGB-LED.

What is commonly known as an RGB-LED is in fact a package containing three small individual LEDs, which emit at the following peak wavelengths: 460 (blue), 525 (green), and 625 nm (red), as shown in Figure 2.

The part highlighted part (Figure 2) corresponds to the so-called “spectral width”, which is defined as the wavelength range that presents an emission intensity greater than/equal to 50% of the maximum value. So, wavelengths located below that threshold have a lower influence on the samples.

As can be observed in Figure 2, each of the LEDs emits certain wavelengths simultaneously. This results means that the water samples will be traversed by more than one wavelength simultaneously, which will cause discrepancies with respect to the results provided by the commercial equipment.

However, commercial equipment based on an incandescent lamps makes use of optical elements, such as monochromators or a diffraction grids, to diffract the light beam in the different wavelengths that make up the source of the light’s spectrum (visible in our case). As a result of this diffraction, a single wavelength is emitted at a time.

The multiple emission spectrums of LEDs have always been one of the reasons why this kind of technology has not become a feasible replacement for the incandescent lamp, as the only way to remove the effect of these “additional wavelengths” was through optical filters, which increase both the size as well as the cost of equipment.

However, through the present research, we defined a simple method that enables the approximation of a single wavelength transmittance by a single RGB-LED without using any optical devices, such as filters of a diffraction grid.

As can already be observed, the emission spectrum of the RGB-LED does not seem to be enough to cover the entire visible spectrum since, as we can see in Figure 2, there are some gaps in certain parts of the RGB-LED spectrum, such as between 546 and 610 nm.

Therefore, our research focused on both: (*i*) Determining what portion of the visible spectrum it is possible to model using each of the individual LEDs that make up the RGB-LED independently, and (*ii*) finding the different combinations of red, green, and blue that produce a response in the samples that has a linear correlation with the transmittance values obtained by spectrophotometric equipment based on incandescent lamps.

### 2.5. Hardware

**Figure 3:**
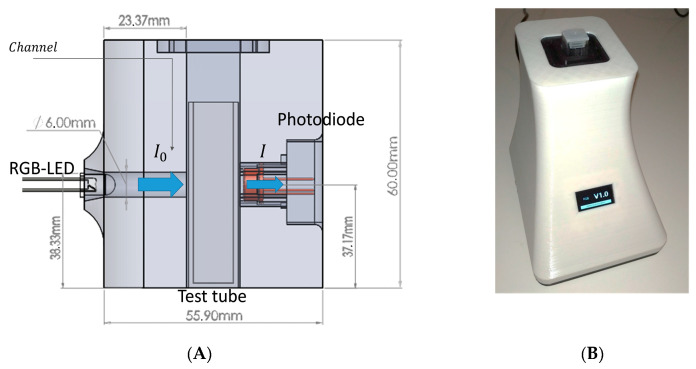
View of the experiment assembly. Schematic view of the assembly. (**A**) Layout diagram of RGB equipment elements. (**B**) Equipment developed for RGB spectrophotometric analysis.

A simplified diagram illustrating the design of the equipment developed is presented in Figure 3. Since one of the objectives was to reduce the size of the device, it was decided not to use optical elements, such as lenses or monochromators, since this is one of the main reasons for the big dimensions of commercial spectrophotometers.

The tests carried out revealed that the best results were obtained when the sensor (Figure 3A right) was as close as possible to the sample without touching it. The light source (Figure 3A left) was at a distance of about 20 mm with reference to the test tube.

### 2.6. Methodology

**Figure 4:**
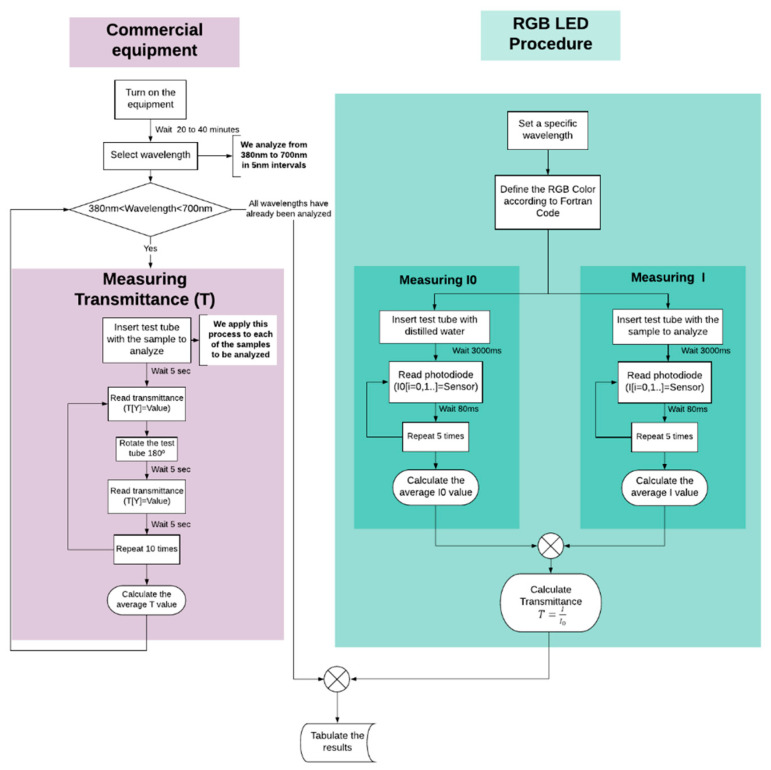
Scheme of the work methodology.

The experimental campaign was followed by a calibration process that will enable the use of the RGB-LED technology as viable to complement incandescent lamps. This process is shown in Figure 4.

## 3. Results and Discussion

### 3.1. Preliminary Tests

The development of spectrophotometers based on RGB-LED has always faced the same issue: This kind of diode cannot emit a wide range of wavelengths (such as the entire visible spectrum); in fact, it only emits three small portions of the spectrum, i.e., 435–485 nm (blue), 500–550 nm (green), and 610–650 nm (red), according to a normal distribution.

**Figure 5:**
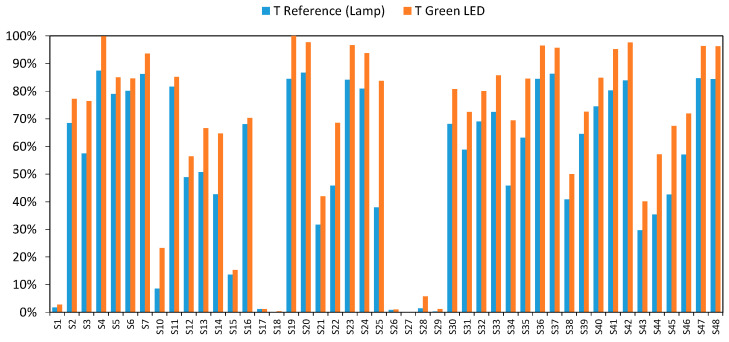
Comparative transmittance value by the green LED and incandescent lamp at 525 nm.

We began our study by analyzing what responses would be obtained when each individual LEDs (red, green, or blue) was turned on separately. Taking the green LED (500 to 550 nm, and 525 nm peak) as an example, Figure 5 shows that the transmission values achieved by the green LED were higher than those provided by the incandescent lamp at 525 nm for the samples detailed in Table 1.

Although the green LED has a peak wavelength of 525 nm, it also emits other wavelengths simultaneously (from 500 to 550 nm). This increases the amount of light that passes through the sample, and consequently, higher than expected transmittance values were obtained.

**Figure 6:**
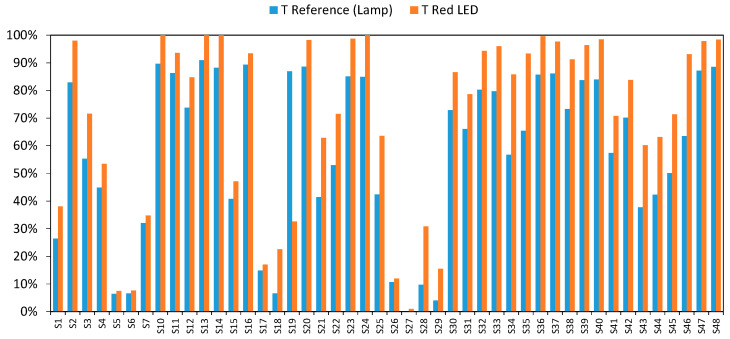
Comparative transmittance value by the red LED and incandescent lamp at 625 nm.

**Figure 7:**
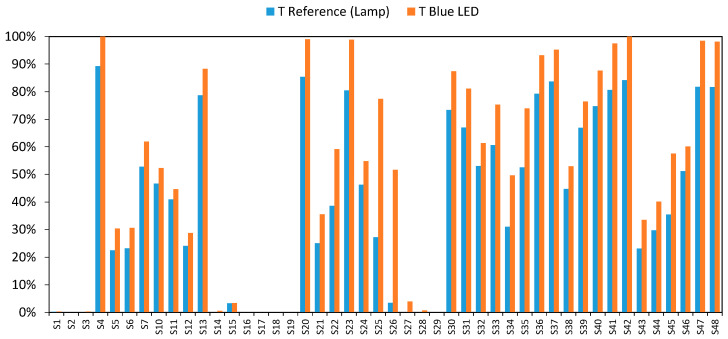
Comparative transmittance values by the blue LED and incandescent lamp at 460 nm.

This behavior is analogous to that observed with the blue and red LEDs (Figure 6 and Figure 7), except that the differences were far more significant in the case of the blue LED.

At this point, it is key to determine how the effect of the remaining wavelengths can be eliminated, without using filters or diffraction grids.

Our investigations concluded that there is a linear relation between the transmittance values provided by the RGB-LED when it uses each of its LEDs (red, green, and blue) independently, and those values that would be obtained with commercial equipment working at the same peak wavelength as the mentioned LEDs.

However, not only can the peak length of each of the LEDs be calibrated but also all the wavelengths emitted by each of them (within a certain range). Nevertheless, we must take into account the fact that as the wavelength that we seek to model moves away from the peak wavelength of each LED, the accuracy of the model is reduced, so not all wavelengths can be successfully modelled.

**Figure 8:**
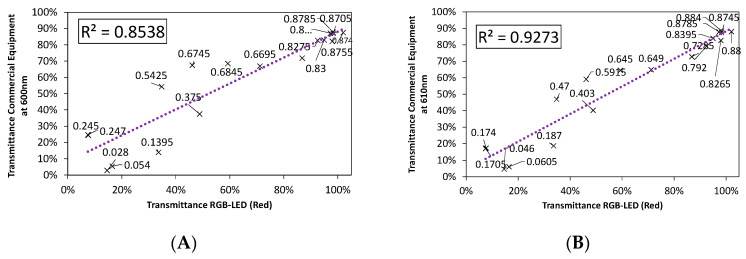

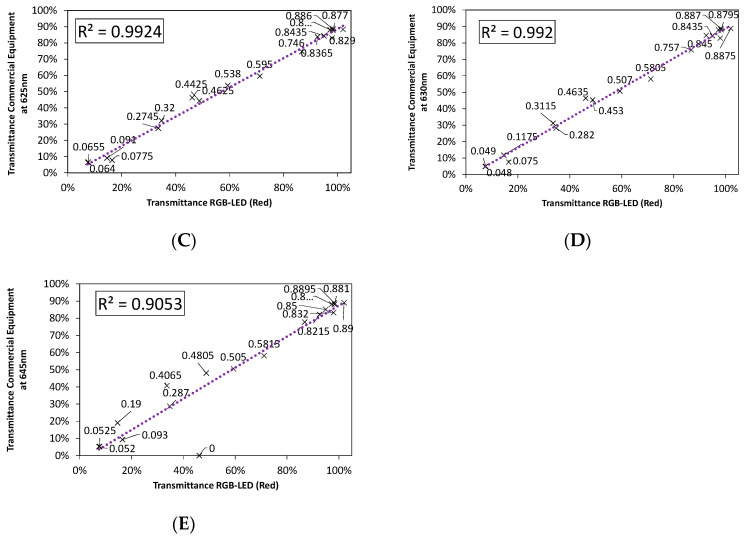
Comparison of the fit for the emission spectrum of the red LED to: (**A**) 600 nm, (**B**) 610, (**C**) 625 nm-λPeak, (**D**) 630 nm, and (**E**) 645 nm.

**Figure 9:**
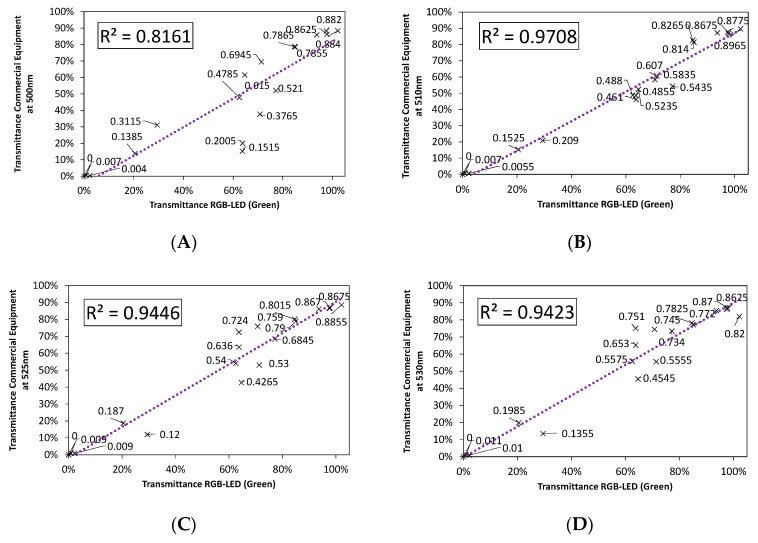

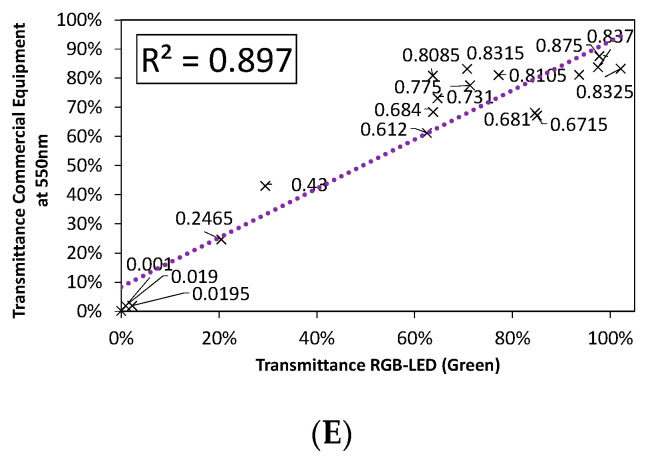
Comparison of the fit for the emission spectrum of the green LED to: (**A**) 500 nm, (**B**) 510, (**C**) 525 nm-λPeak, (**D**) 530 nm, and (**E**) 550 nm.

**Figure 10:**
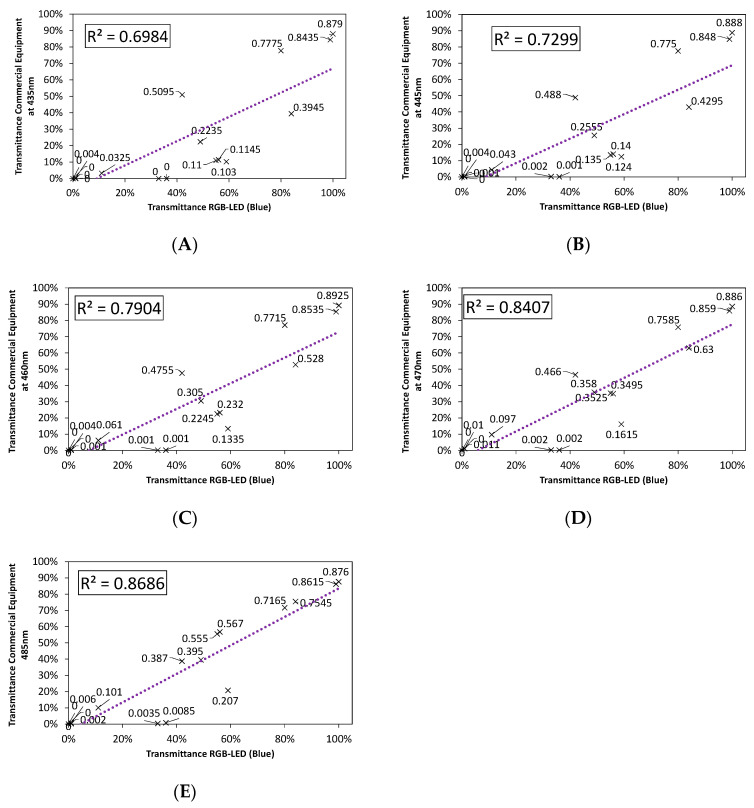
Comparison of the fit for the emission spectrum of the Blue LED to: (**A**) 435 nm, (**B**) 445 nm, (**C**) 460 nm-λPeak, (**D**) 470 nm, and (**E**) 485 nm.

In order to determine which portion of the emission spectrum in each of the LEDs can be used for spectrophotometric applications, Figure 8, Figure 9 and Figure 10 show the fit between the transmittance measured from incandescent lamps and that from red, green, and blue RGB-LEDs, independently, for five selected wavelengths. These figures were calculated using the S1–S20 samples shown in Table 1.

It can be observed how the accuracy of the fit varies, improving when the wavelength approaches the peak of each LED.

As we can see in Figure 8, Figure 9 and Figure 10, the red LED showed the best performance, with a goodness-of-fit above 90%, closely followed by the green LED. On the other hand, the blue LED presented the largest disparity, since its fit was quite low, it is therefore not suitable for spectrophotometry applications. As expected, the best fits were obtained at the peak wavelength and decreased as we moved away from that point.

**Figure 11:**
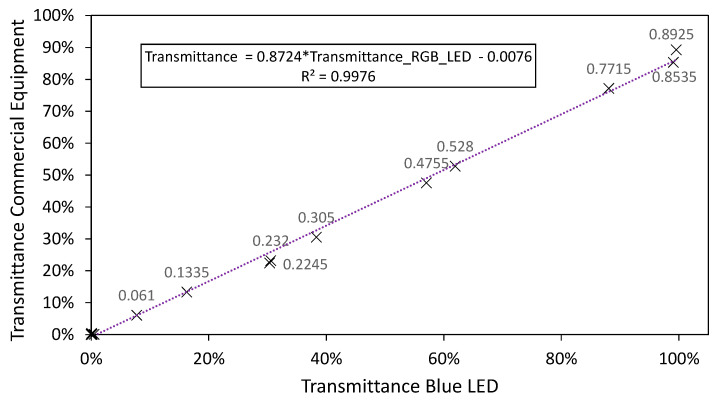
Fit of the LED whose peak wavelength is 460 nm.

In order to understand the low performance of the RGB blue LED, Figure 11 shows the transmittance correlation of a single blue LED, with a peak wavelength of 460 nm, which does not belong to an RGB LED but coincides with its peak wavelength (Figure 10D).

As can be seen, the differences are noteworthy. The single blue LED produces a much better fit (higher than 99%) in comparison with the RGB-LED when only blue is selected (79%) as shown in Figure 10C.

The test carried out concluded that these results were due to the low-quality semiconductor used in most RGB LEDs. An LED emits light as a result of the jump (bandgap) of electrons when they try to pass from one layer (type N) to another where there are holes (type P). The size of this bandgap will determine the type of light emitted by the electrons. A small bandgap will give rise to red light (around 1.91 eV), and a large bandgap to blue light (2.64 eV) [42,43]. The greater the bandgap, the bluer the light emitted by the diode.

Most blue individual LEDs, such as in Figure 11, are based on a 1-µm sapphire substrate, on which alternate layers of gallium nitrate, indium, and aluminum are grown. These extra elements are key to increasing the efficiency and brightness of the blue LEDs. Moreover, with the use of aluminum, it is possible to make even ultraviolet LEDs.

It is necessary to mention that generating blue LEDs has been a technological challenge for years. In fact, Isamu Akasaki, Hiroshi Amano, and Shuji Nakamura’s research on the development of this type of diode won them the Nobel Prize in Physics in 2014 [44].

The issue is that RGB-LEDs are designed to be used for visual purposes, where the combination of primary colors creates the illusion that certain colors are being generated. This optical effect means that it is not necessary to design RGB-LEDs that generate a “pure blue”, but something that our eye perceives as blue [45] (in combination with red and green). For this reason, the quality of the substrates used and the purity of the crystals are lower than the individual LEDs (Figure 11). Proof of this is that an RGB-LED is around 10 times cheaper than an individual one.

**Figure 12:**
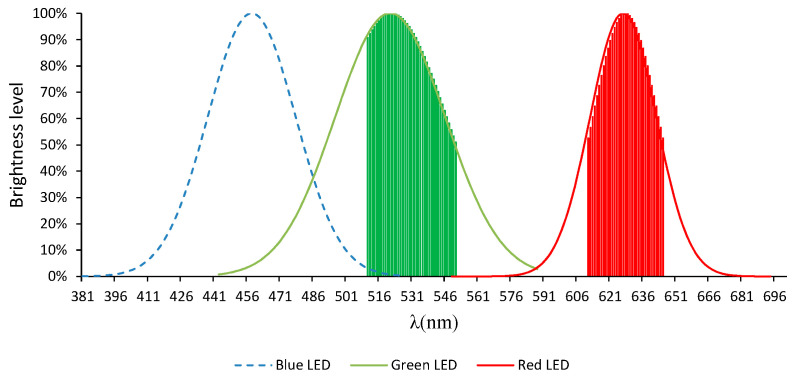
Useful emission spectrum of the RGB-LED.

Therefore, although an RGB-LED makes use of three different individual LEDs, which emit in a large portion of the visible spectrum, only the red and green LEDs seem to be suitable. Moreover, of these, only a portion of the spectrum between the following wavelengths is suitable for spectrophotometric applications: 510 to 550 nm (green LED) and 610 to 645 nm (red LED), as presented in Figure 12.

To be able to perform analyses outside that range, for example, between 380 and 500 nm, the RGB-LED would not be sufficient, and therefore, it should be combined with an individual blue LED (with a peak wavelength of 460 nm) to replace the one already present on the RGB-LED. This individual LED would allow measurements outside the range of 510–645 nm as shown in Figure 11.

However, in this research work, we only focused on the maximum spectral range that could be modelled using a single RGB-LED, in order to minimize the number of components needed to develop a spectrophotometry equipment.

### 3.2. Extension of the Working Range

In the previous section, it was shown that the red and green LEDs can model a reduced wavelength of the visible spectrum (Figure 12). It will now be studied if certain combinations of red and green can produce a response in the samples that has a linear correlation with the transmittance values that would be obtained with an incandescent lamp.

The transmittance value of a water sample is the response of the physical and chemical properties of the solids present in the water to a certain wavelength. The mere combination of three or two different colors (groups of wavelengths) will never provide the same spectral response that a certain wavelength would achieve. That is to say, you cannot create artificial wavelengths by simply combining colors, even though the human eye perceives the opposite. For instance, the combination of blue and yellow produces green, but this does not mean that the wavelengths from 500 to 550 nm are being emitted, it is just an optical effect [45].

#### 3.2.1. Color Rendering

**Figure 13:**
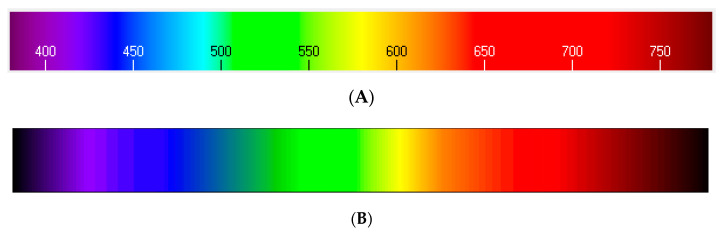
(**A**) Comparative visible spectrum based on RGB equivalence equations- λ. (**B**) Real visible spectrum.

For this reason, a color rendering method was implemented, specifically, an algorithm developed by Dan Bruton of the Texas A&M University [46], which enables the approximation of a specific RGB combination to a certain color from a visual point of view, as Figure 13 shows.

This will allow us to obtain the correlations, in terms of transmittance, between the light wavelengths contained in the range from 380 to 700 nm and the RGB values, from the point of view of color representation. We observed that those color combinations (red, green, and blue) that are visually closer to the wavelength we seek to model (Figure 13A) produce transmittance values that reach a linear correlation with the transmittance values obtained with commercial equipment based on incandescent lamps (Figure 13B). Figure 13A was obtained from the RGB combinations provided by the equations that will be shown in this section.

The determination of the correlation between RGB and the wavelength Lambda (λ) is carried out by several sets of equations grouped by spectral regions, as presented in Equation (1), where R′, $G^{\prime },$ and B′ are the red, green, and blue adopted values in the 0–1 range, respectively, without considering the brightness level Y:

(1) $$\left(380\;\mathrm{nm}\leq \;\lambda <440\;\mathrm{nm}\right)\to \{\begin{array}{c} R\prime =-\left(\frac{\lambda -440}{440-380}\right)\\ G\prime =0\\ B\prime =1 \end{array}\;\left(440\;\mathrm{nm}\leq \;\lambda <490\;\mathrm{nm}\right)\to \{\begin{array}{c} R\prime =0\\ G\prime =\left(\frac{\lambda -440}{490-440}\right)\\ B\prime =1 \end{array}\;\left(490\;\mathrm{nm}\leq \;\lambda <510\;\mathrm{nm}\right)\to \{\begin{array}{c} R\prime =0\\ G\prime =1\\ B\prime =-\left(\frac{\lambda -510}{510-490}\right) \end{array}\;\left(510\;\mathrm{nm}\leq \;\lambda <580\;\mathrm{nm}\right)\to \{\begin{array}{c} R\prime =\left(\frac{\lambda -510}{580-510}\right)\\ G\prime =1\\ B\prime =0 \end{array}\;\left(580\;\mathrm{nm}\leq \;\lambda <645\;\mathrm{nm}\right)\to \{\begin{array}{c} R\prime =1\\ G\prime =-\left(\frac{\lambda -645}{645-580}\right)\\ B\prime =0 \end{array}\;\left(645\;\mathrm{nm}\leq \;\lambda <781\;\mathrm{nm}\right)\to \{\begin{array}{c} R\prime =1\\ G\prime =0\\ B\prime =0 \end{array}.$$

The brightness level Y is another factor to take into account when calculating correlations. This is defined according to the spectrum that we wish to calculate (Equation (2)):

(2) $$\left(380\;\mathrm{nm}\leq \;\lambda <420\;\mathrm{nm}\right)\to Y=0.3+0.7*\frac{\left(\lambda -380\right)}{420-380}\;\left(420\;\mathrm{nm}\leq \;\lambda <701\;\mathrm{nm}\right)\to Y=1\;\left(701\;\mathrm{nm}\leq \;\lambda <781\;\mathrm{nm}\right)\to Y=0.3+0.7*\frac{\left(780-\lambda \right)}{780-700}.$$

The RGB value is calculated through two additional parameters: Gamma=0.8. To be able to perform the current variation, we used an 8-bit driver, which can provide $2^{8}-1=256$ different values, i.e., between 0 and 255, and therefore, the maximum working range that the LED will operate is I = 255. However, this procedure would be scalable to other resolutions. For example, a 10-bit current driver would make I take the value of 1023.

Finally, the expressions that allow the determination of the correlation between λ and the RGB value are shown in Equation (3):

(3) $$\{\begin{array}{c} \left(R\prime =0\right)\to R=0\\ \left(R\prime \neq 0\right)\to R=I*{\left(R\prime *Y\right)}^{Gamma} \end{array}\;\{\begin{array}{c} \left(G\prime =0\right)\to G=0\\ \left(G\prime \neq 0\right)\to G=I*{\left(G\prime *Y\right)}^{Gamma} \end{array}\;\{\begin{array}{c} \left(B\prime =0\right)\to B=0\\ \left(B\prime \neq 0\right)\to B=I*{\left(B\prime *Y\right)}^{Gamma} \end{array},$$

where R, G, and B are the current values required to be able to achieve a specific color through an RGB-LED.

**Figure 14:**
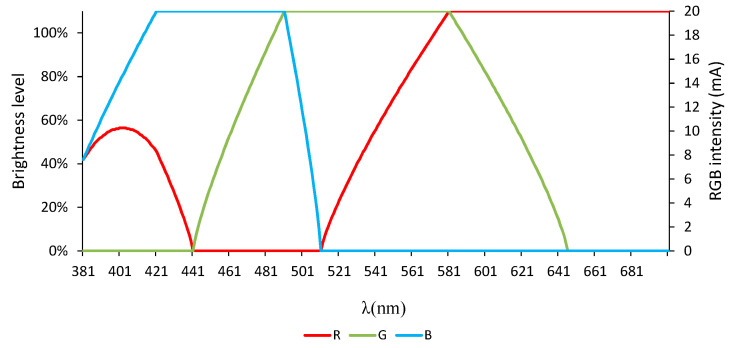
Heuristic RGB values for visible wavelengths by color rendering.

The variation of the RGB values to visually achieve the wavelengths between 380 and 700 nm, from the previous Equations (1)–(3), is shown in Figure 14. The working range of each RGB-LED can take a value between 0 and 255. However, in order to clarify the exposure of the results, the brightness level given in Figure 14 was expressed as a function of the current applied to each LED, in mA, taking into account that the highest level of intensity of the LED (255) is reached at 20 mA.

#### 3.2.2. LED Combination Calibration

As was previously stated in Section 3.1, the red and green LEDs are able to explain by themselves (without combining them) the wavelength ranges between 510 and 550 nm and 610 and 645 nm with a high accuracy. However, they cannot model other parts of the visible spectrum. Therefore, in this section, we focus the study on the following three parts of the visible spectrum, which have not been modelled by red and green LED themselves: 550 to 610 nm, 645 to 700 nm, and 380 to 550 nm.

To analyze the behavior of the samples in these regions, the lighting brightness level of each LED (amount of current applied, which is directly proportional to the level of brightness) was set to produce the same color as the wavelength we sought to model using the color rendering algorithm presented in Figure 14 through Equations (1)–(3).

As commented, the combination of RGB-LEDs to achieve the visual effect of a defined wavelength will not generate the transmittance response expected of an incandescent lamp in the studied wavelength. However, we observed that wavelength combinations that produce (from the visual point of view) a color closer to the one we want to model (within a certain spectrum band) produce a response in the samples that is linearly related to the transmittance values that an incandescent lamp would provide at the same wavelength.

The tests carried out proved that wavelengths between 550 and 610 nm can be adjusted with the combination of LEDs according these equations.

**Figure 15:**
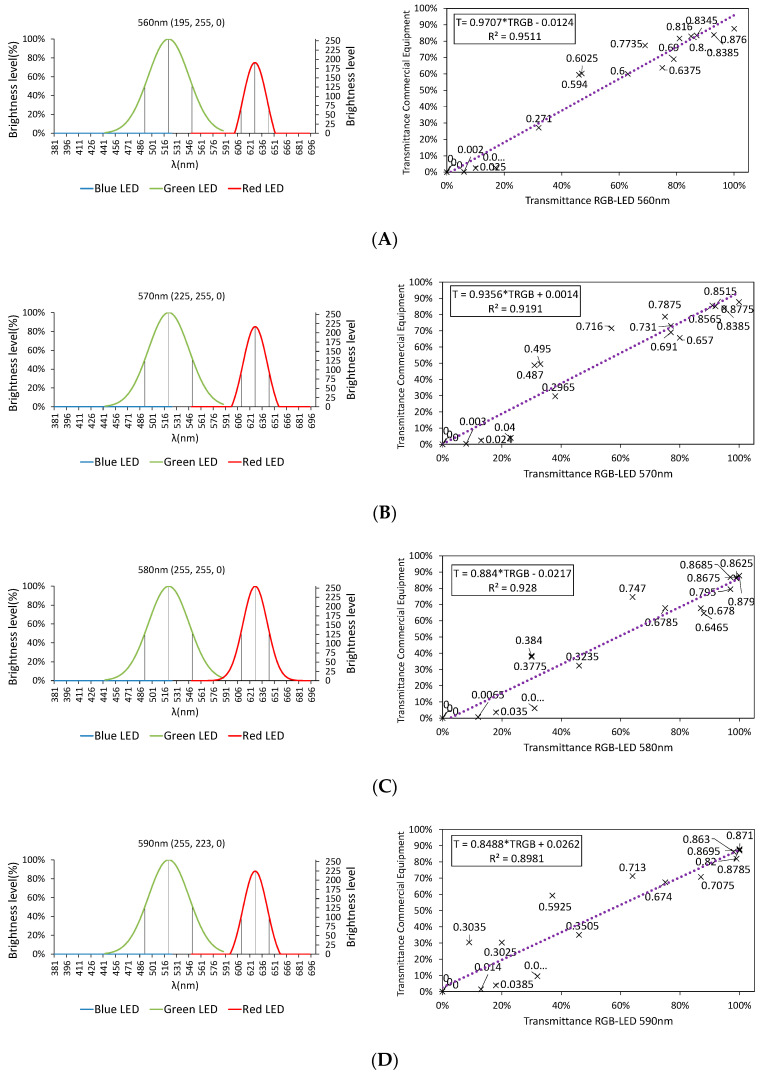

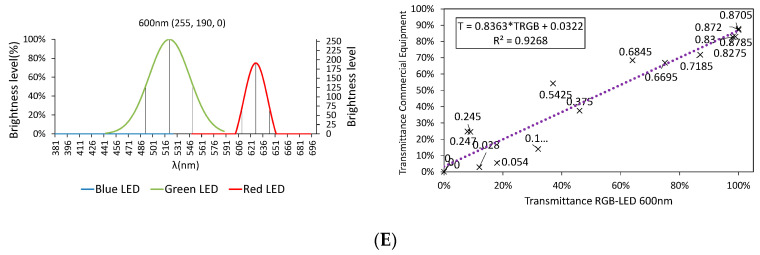
Combination of wavelengths and correlation for: (**A**) 560, (**B**) 570, (**C**) 580, (**D**) 590, and (**E**) 600 nm.

Figure 15 shows, the emission spectrum to render the different wavelengths (according to the color rendering algorithm shown in Section 3.2.1) on the left, and the correlation between the transmittance values provided by such a combination of LEDs and those that would be obtained with commercial equipment based on incandescent lamps, for the following wavelengths: 560, 570, 580, 590, and 600 nm and for the 20 samples designed in Table 1 for the calibration process (S1–S20), on the right.

**Figure 16:**
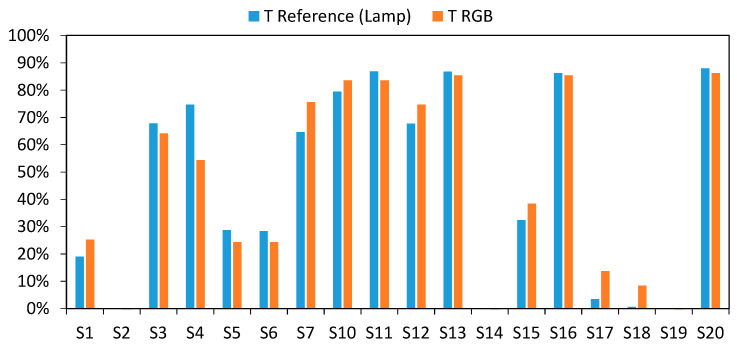
Comparative transmittance value by the RGB-LED and incandescent lamp at 580 nm.

As can be observed, all the cases provide a fit higher than 90%. Figure 16 shows the comparison between the transmittance values obtained by the RGB-LED and the incandescent lamp at 580 nm for the samples included in Figure 15.

Although the LEDs are not emitting wavelengths between 560 and 600 nm, the combination of their wavelengths is able to produce a response in the samples that can be related to the transmittance values expected in commercial equipment.

**Figure 17:**
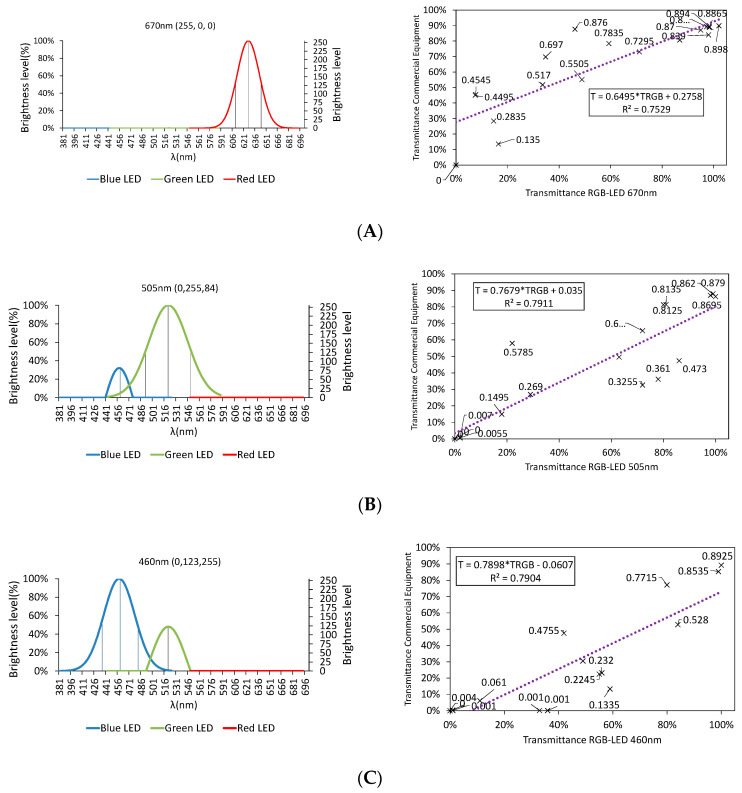
Combination of wavelengths and correlation for: (**A**) 670, (**B**) 505, and (**C**) 460 nm.

However, the remaining wavelengths (greater than 645 nm and less than 510 nm) could not be modelled with the LEDs working individually, nor indeed with any combination of them. Figure 17 shows that the goodness-of-fit was less than 80%, thus rendering them unsuitable for the development of spectrophotometry equipment.

In order to understand this behavior, we performed the analysis of any three wavelengths, taken in different color regions: 670, 505, and 460 nm; the results are shown in Figure 17.

As we can see, the goodness-of-fit is lower than 80% in all cases. In 670 nm (Figure 17A), the emission diagram shows that only the red LED is on, i.e., there is no combination of wavelengths but, despite this, as 670 nm is outside the spectral width of the red LED (Figure 12), the correlation is very poor.

Something different seems to explain the lack of fit accuracy in the range from 460 to 505 nm. In this range, there is a combination of green and blue LEDs. Although the wavelength is in the spectral width covered by green and blue LEDs, the presence of the blue LED weighs down the results, due to the low quality of its materials. Therefore, we can conclude that a single RGB-LED can model only the spectrum between 510 and 645 nm, with a goodness-of-fit above 90%, without using any optical device.

**Figure 18:**
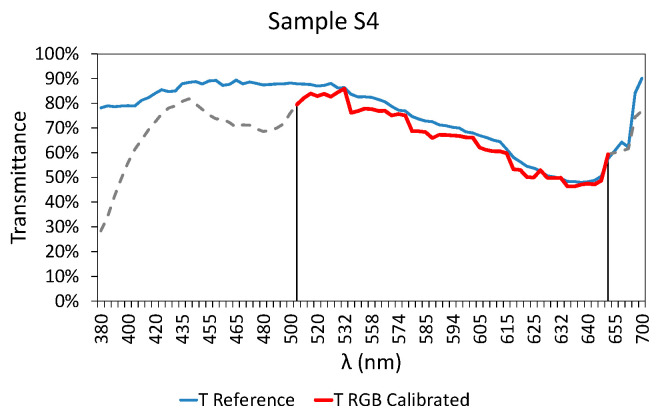
RGB-LED calibration between 380 and 700 nm for sample S4.

Figure 18 shows a comparison between the transmittance values obtained for sample S4 using an incandescent lamp and the transmittance values obtained with the RGB-LED after the calibration process. The region between 510 and 645 nm (which is where the RGB-LED has shown the best performance) was highlighted in red to facilitate the reader’s understanding. Additionally, the other parts of the spectrum from RGB-LED, which could not be modelled correctly, are shown in grey. It can be observed that the fit is quite good in the designated portion of the spectrum.

At this point, it is important to note that other non-linear models have been tested without success. Only linear regression models have been able to determine the correlation between RGB combinations and transmittance values provided by commercial equipment with a high precision.

### 3.3. Final Results

**Figure 19:**
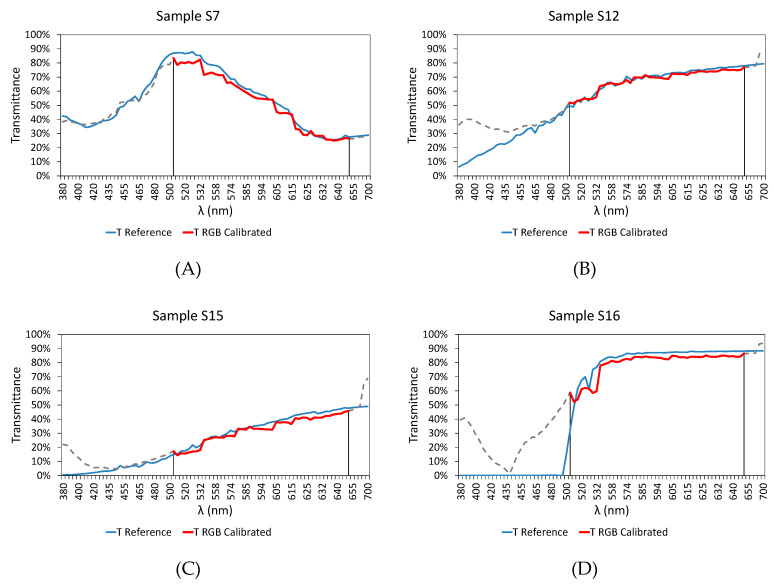

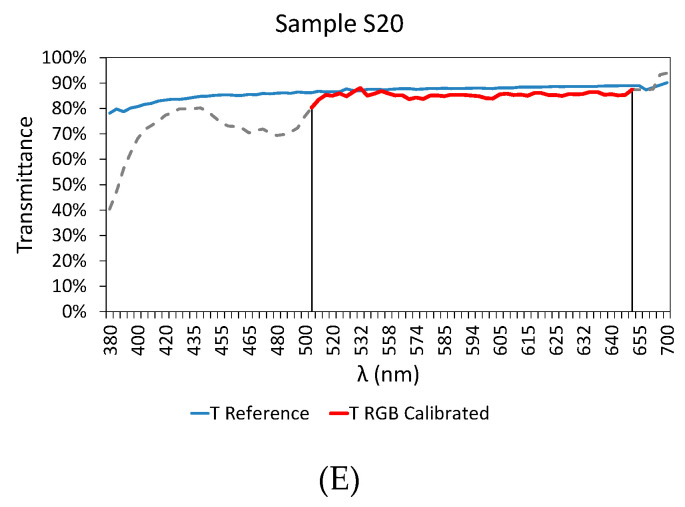
Comparative results of the transmittance values between commercial equipment based on the incandescent lamp (blue) and RGB-LED device developed (red) after the calibration within 510–645 nm, for the following samples used during the calibration process: (**A**) S7, (**B**) S12, (**C**) S15, (**D**) S16, and (**E**) S20.

**Figure 20:**
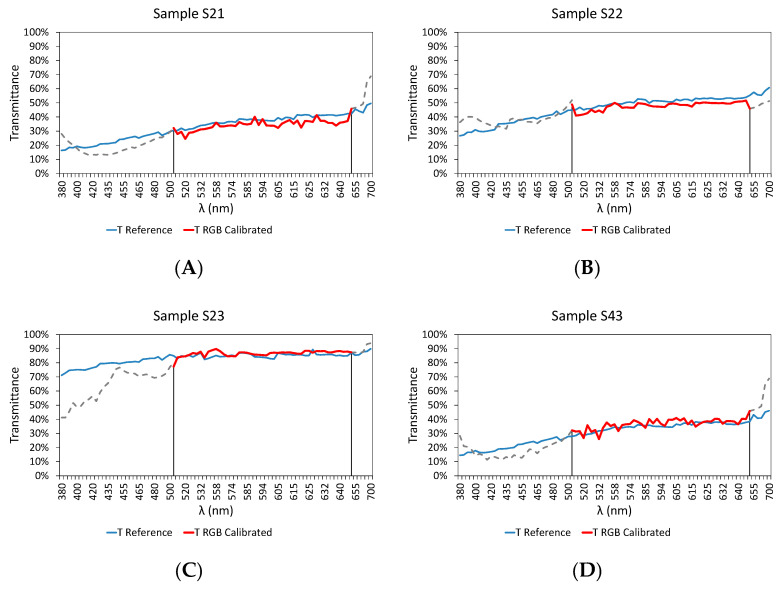

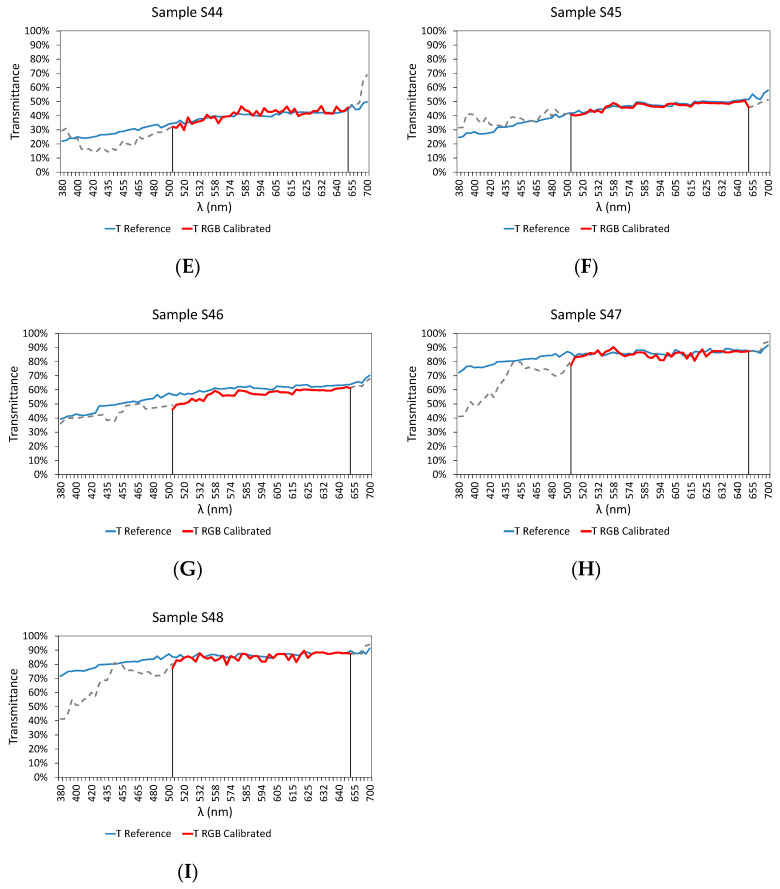
Comparative results of the transmittance values between commercial equipment based on the incandescent lamp (blue) and RGB-LED device developed (red) after the calibration within 510–645 nm, for the following samples not used for the calibration process: (**A**) S21, (**B**) S22, (**C**) S23, (**D**) S43, (**E**) S44, (**F**) S45, (**G**) S46, (**H**) S47, and (**I**) S48.

In order to show the suitability of the calibration models, the results of the analysis carried out in the range of 510 to 645 nm are shown for different samples. To clarify the presentation of the results, we distinguished between those samples used during the calibration process (Figure 19) and those used to check the effectiveness of the models (Figure 20), i.e., samples that were not used during the calibration process (Table 1 from S21 to S48).

As can be seen, the charts shown in Figure 19 how a good level of fit within the 510–645 nm zone. Outside that range, the transmittance values differ from those provided by the reference equipment (incandescent lamp).

However, in order to show the suitability of the model with unused samples during the calibration process, Figure 20 shows the results obtained with wastewater samples (S21–S23 and S43–S48), corresponding to different points of a wastewater treatment plant, specifically: Wastewater treatment plant inlet (S21, S43, and S44), at the Primary Settler outlet (S22, S45, and S46) and at the treatment plant outlet (S23, S47, and S48).

As can be observed in Figure 20, all the samples analyzed show a high accuracy according to the transmittance values provided by the commercial equipment based on incandescent lamps within 510 nm to 645 nm.

Table 2, provides the reader with further information regarding the kind of wastewater the RGB-LED is valid for, settler showing the main properties of wastewater samples analyzed in Figure 20, namely: Chemical oxygen demand (COD), biological oxygen demand at 5 days (BOD5), total suspended solids (TSSs), phosphorus (P), nitrate nitrogen (NO3-N), pH, and conductivity.

One of the main characteristics of urban wastewater is the existence of different suspended substances, which influence the amount of light that passes through the samples.

A key issue in determining the validity of the RGB-LED was whether the presence of such suspended particles would have a different effect on the transmittance values measured with the RGB-LED than with equipment based on incandescent lamps.

The tests carried out showed that the spectral response within the range of 510–645 nm provided by the RGB-LED has been close to that of the reference equipment. Samples S21, S43, and S44, which had the highest concentration of total suspended solids (SST) provided proof of that.

Therefore, we conclude that these suspended particles affect both instruments in the same way, highlighting the validity of the RGB-LED for the analysis of wastewater.

Although RGB-LED is not able to model the whole visible spectrum, it provides a simple method to carry out a spectrophotometric analysis without the use of optical elements.

In order to quantify the precision of the results provided by RGB-LED after the calibration process, several statistical indicators were calculated: The Root-Mean-Square Deviation (RMSD) [46], and the error index, Er, through Equations (4) and (5):

(4) $$RMSD=\sqrt{\frac{1}{n}\sum_{i}^{n}{\left(T_{measured\_i}-T_{calculated\_i}\right)}^{2}},$$

(5) $$Er\left(\%\right)=\frac{\sum_{i}^{n}\left(T_{measured\_i}-T_{calculated\_i}\right)}{\sum_{i}^{n}T_{measured\_i}}*100,$$

where *Er* is the error index (%); *n* is the number; and $T_{measured}$ and $T_{calculated}$ are the transmittance values obtained through the commercial equipment [32] and our design based on RGB-LED technology, respectively.

Table 3 shows both the error index and the RMSD value from the samples shown in Figure 19 (used during the calibration process) and Figure 20 (test samples of wastewater), only for the spectrum between 510 and 645 nm.

For all the samples, the error level was always been less than 6%, including in the case of wastewater samples from urban areas. This proves the RGB-LED technology can be used for spectrophotometric applications, achieving a fairly close response to the commercial equipment, and enabling the development of more cost-effective equipment.

## 4. Conclusions

In this paper, we studied the possibility of using an RGB-LED device to work as a spectrophotometer to calculate transmittance. This is a cost-effective and robust piece of equipment that can be used as a complement to traditional incandescent lamps in the 510 to 645 nm range.

The method proposed in this research work is based on an RGB combination to produce a chemical response in water samples, which can be correlated with the expected transmittance values obtained with commercial equipment based on incandescent lamps, where a single wavelength passes through the sample.

The calibration procedure enables us to achieve results with an RGB-LED that are comparable to those highly accurate results obtained. Proof of this lies in the fact that the error obtained was less than 6% in all the cases (Table 3).

The methodology proposed:

(i) Demonstrates that RGB-LED can be used to carry out a spectral analysis of wastewater, obtaining results very close to those provided by commercial equipment based on incandescent lamps.
(ii) Develops a calibration system for measuring transmittance values between 510 and 645 nm using a single RGB-LED, with high accuracy. Moreover, it enables us to reduce the number of elements used, and therefore, significantly reduce the cost of the equipment.
(iii) Models the transmittance value of a specific wavelength without the need for optical elements, such as monochromators or diffraction gratings.
(iv) Uses the red and green LEDs in combination to model those parts of the visible spectrum that cannot be modelled by the RGB-LED when each of the LEDs that composes it acts individually. This allows the wavelength range to be extended without increasing the number of LEDs used.
(v) Achieves reductions in the dimensions, costs, and sampling times of the equipment, which are vital aspects for the development of low-cost autonomous systems designed to measure in any type of environment.

This calibration procedure can serve as help when developing one’s own spectrophotometry equipment. This research contributes to the development of cost-effective equipment.

A model valid for the measured samples was proposed, where the modelled spectrum transmittance adjusts to that values calculated with commercial equipment between 510 and 645 nm by using a single RGB-LED through a color rendering algorithm, without optical elements.

Although it is not possible to use RGB technology to develop full visible spectrum spectrophotometry equipment (380–700 nm), the range of 510 to 645 nm could be of help to characterize certain parameters, such as chlorophyll types C2 and C3 [45], for instance.

This research can help in the development of new systems based on this technology, lowering the cost of equipment whilst also reducing its size and consumption, thus enabling the creation of autonomous equipment that can run on batteries in any environment.

This type of system can be very useful for the detection of unauthorized discharges, as the system can be placed in any environment, monitoring 24 h a day. It is therefore a tool to combat fraud and can contribute to environmental protection.

All this research work will allow us to have real-time control of water quality in sewer systems during rainfall events and dry weather periods, through a portable and cost-effective device to analyze the contaminant load present in wastewater.

## Figures and Tables

**Table 1:** Analyzed samples.

	Designation	Substance	Dissolution
**Calibration Samples**	S0	Distilled water	100%
	S1	Red wine	50%
	S2	Tea	80%
	S3	Yellow and Blue food dye	20%–80%
	S4	Blue food dye	50%
	S5	Washing machine detergent	50%
	S6	Washing machine detergent	65%
	S7	Washing machine detergent	75%
	S8	Milk	100%
	S9	Milk	50%
	S10	Red food	50%
	S11	Kitchen oil	100%
	S12	Vinegar	90%
	S13	Red food	75%
	S14	Red food	55%
	S15	Soluble coffee	75%
	S16	Yellow food dye	40%
	S17	Soluble coffee	50%
	S18	Red wine	100%
	S19	Blue food dye	30%
	S20	Sea water	100%
**Test Samples**	S21	Urban wastewater Wastewater treatment plant inlet	100%
	S22	Urban wastewater Primary settler	100%
	S23	Treated wastewater Wastewater treatment plant outlet	100%
	S24	Olive oil	100%
	S25	Cocoa powder	5%
	S26	Cocoa powder	30%
	S27	Cocoa powder	55%
	S28	Caffeine powder	10%
	S29	Caffeine powder	30%
	S30	Cetylpyridinium chloride	50%
	S31	Cetylpyridinium chloride	100%
	S32	Beer	100%
	S33	Beer	50%
	S34	Olive water	100%
	S35	Olive water	50%
	S36	White wine	100%
	S37	White wine	50%
	S38	Pinkish visage	100%
	S39	Pinkish visage	50%
	S40	Pinkish visage	30%
	S41	Amphoteric surfactants	100%
	S42	Amphoteric surfactants	50%
	S43	Urban wastewater WWTP inlet	100%
	S44	Urban wastewater WWTP inlet	100%
	S45	Urban wastewaterWWTP Primary settler	100%
	S46	Urban wastewaterWWTP Primary settler	100%
	S47	Treated wastewater WWTP outlet	100%
	S48	Treated wastewater WWTP outlet	100%

**Table 2:** Characteristics of wastewater shown in Figure 20.

**Polluting Parameters**	**Wastewater Inflow** **(S21)**	**Primary Settler** **(S22)**	**Treated Water** **(S23)**
COD	763 mg/L	475 mg/L	52 mg/L
BOD5	500 mg/L	310 mg/L	9 mg/L
TSS	304 mg/L	88 mg/L	14 mg/L
Phosphorus (P)	9.1 mg/L	7.2 mg/L	2.5 mg/L
Total Nitrogen (TN)	74 mg/L	74 mg/L	16.6 mg/L
NO3-N	0.5 mg/L	0.3 mg/L	10.3 mg/L
PH	7.59	7.5	7.56
Conductivity	2770 µS/cm	2590 µS/cm	2580 µS/cm
	**Wastewater Inflow** **(S43)**	**Wastewater Inflow** **(S44)**	**Primary Settler** **(S45)**
COD	1275 mg/L	908 mg/L	727 mg/L
BOD5	720 mg/L	480 mg/L	460 mg/L
TSS	624 mg/L	558 mg/L	151 mg/L
Phosphorus (P)	8.7 mg/L	9.4 mg/L	12.5 mg/L
Total Nitrogen (TN)	75 mg/L	59 mg/L	89 mg/L
NO3-N	0.6 mg/L	0.8 mg/L	0.3 mg/L
PH	7.48	7.14	7.24
Conductivity	2590 µS/cm	2630 µS/cm	2600 µS/cm
	**Primary Settler** **(S46)**	**Treated Water** **(S47)**	**Treated Water** **(S48)**
COD	732 mg/L	47 mg/L	46 mg/L
BOD5	470 mg/L	6 mg/L	5 mg/L
TSS	106 mg/L	11 mg/L	13 mg/L
Phosphorus (P)	12.2 mg/L	0.8 mg/L	1.4 mg/L
Total Nitrogen (TN)	73 mg/L	59 mg/L	19.3 mg/L
NO3-N	0.5 mg/L	3.8 mg/L	10.8 mg/L
PH	7.13	7.69	7.45
Conductivity	2930 µS/cm	2340 µS/cm	2170 µS/cm

**Table 3:** Root-Mean-Square Deviation (RMSD) and error index 510–645 nm.

	Sample	RMSD	Er (%)
Calibration Samples	S4	0.038	2.453
	S7	0.042	3.415
	S12	0.017	0.920
	S15	0.028	5.252
	S16	0.051	4.173
	S20	0.028	1.691
Test Samples	S21	0.038	5.688
	S22	0.031	3.595
	S23	0.0231	−1.125
	S43	0.0297	−3.163
	S44	0.0257	−1.009
	S45	0.0125	0.8946
	S46	0.0408	3.7038
	S47	0.0217	0.5203
	S48	0.0222	0.7365
